# Pipeline Embolization Device for Salvage Treatment of a Willis Covered Stent Prolapse Into the Aneurysmal Sac

**DOI:** 10.3389/fneur.2019.01099

**Published:** 2019-10-17

**Authors:** Zeng-Bao Wu, Sheng Wang, Li-Gang Song, Xin-Jian Yang, Shi-Qing Mu

**Affiliations:** ^1^Department of Neurosurgery, Tongji Hospital, Tongji Medical College, Huazhong University of Science and Technology, Wuhan, China; ^2^Department of Interventional Neuroradiology, Beijing Tiantan Hospital, Capital Medical University, Beijing, China

**Keywords:** endoleak, migration, prolapse, pipeline embolization device, Willis covered stent

## Abstract

The Willis covered stent (WCS) may prolapse into the aneurysmal sac due to device migration or foreshortening. We present a useful salvage strategy that can reorient a prolapsed WCS into a more suitable alignment. An intra-procedural prolapse of a WCS into a large cavernous aneurysm occurred in a 70-year-old female patient. A pipeline embolization device (PED) was used to retrieve the WCS and successfully accomplish flow diversion. Maintaining proximal access and ensuring that the microwire is securely held within the central axis of the herniated stent are critical until the entire parent vessel can be reconstructed. This salvage technique may help to regain proximal access and reposition the flow diversion constructs following WCS prolapse.

## Background

The Willis covered stent (WCS, MicroPort, Shanghai, China) is approved for the treatment of intracranial aneurysms including distal internal carotid artery (ICA) aneurysms, recurrent intracranial aneurysms after coiling, fusiform carotid aneurysms, and large or giant intracranial aneurysms, and it has yielded good outcomes (1–5). However, rare intra-procedural complications during WCS deployment have been reported, especially complications associated with device migration. Stent migration can result in device herniation into the aneurysm sac, as well as incomplete neck coverage, which can lead to serious complications including vessel thrombus, aneurysm rupture, endothelial damage during retrieval, or dislodging of embolic material with subsequent stroke (6–9). Compared with incomplete neck coverage, stent prolapse into the aneurysm sac is more problematic because of the difficulty associated with regaining distal or proximal access to reconstruct the parent artery.

The pipeline embolization device (PED, Medtronic-Covidien Neurovascular, Irvine, CA, USA) is a safe and effective treatment for recurrent intracranial aneurysms following clipping (10), coiling (11), and stent-assisted embolization (8, 12); however, its utility in the management of aneurysms previously treated with a WCS that migrated and prolapsed into the aneurysm sac has not been reported. Here we describe the first use of a PED to realign proximal access to the previous WCS for secondary reconstruction of the parent artery.

## Case Presentation

### WCS Deployment and Lost Access

A 70-year-old female presented with a right oculomotor nerve palsy and was found to have a large right cavernous ICA aneurysm (12 × 10 mm, 12-mm neck). Due to the location and size, endovascular treatment with a WCS was recommended.

**Figure 1:**
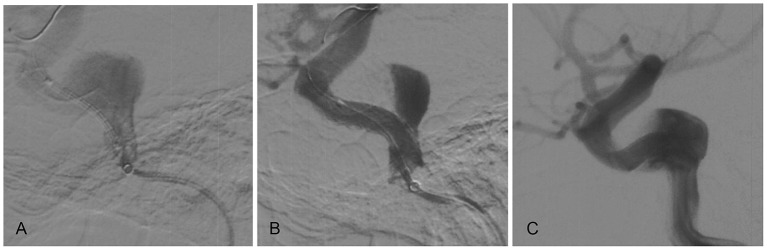
The WCS prolapsed into the aneurysm. **(A)** A WCS bridged the aneurysm neck under roadmap guidance. **(B)** Control angiography showed a mild endoleak after WCS placement. **(C)** Distal migration of the graft occurred with balloon removal, and the proximal part of the stent prolapsed into the aneurysm.

We placed a Rebar microcatheter (Medtronic-Covidien Neurovascular) inside a 5-French Navien (Medtronic-Covidien Neurovascular) so that the Navien could be carried into the horizontal segment of the right petrous ICA, thus facilitating subsequent WCS deployment. The Rebar microcatheter was withdrawn. Using roadmap guidance, a WCS measuring 4.5 × 16.0 mm was bridged into the aneurysm neck (Figure 1A). Balloon deflation was performed with 6 atm pressure under fluoroscopic control. After that, a mild proximal endoleak was observed (Figure 1B). The balloon was slowly re-inflated with 10 atm pressure, but the leak persisted. Worse still, distal migration of the graft occurred upon balloon removal, which led to the proximal part of the stent prolapsing into the aneurysm (Figure 1C). An additional WCS was used to overlap the previous one, but this failed. The operation was stopped, and the patient was transferred to our hospital for treatment.

### Anterograde PED-Assisted Rescue of Lost Access

**Figure 2:**
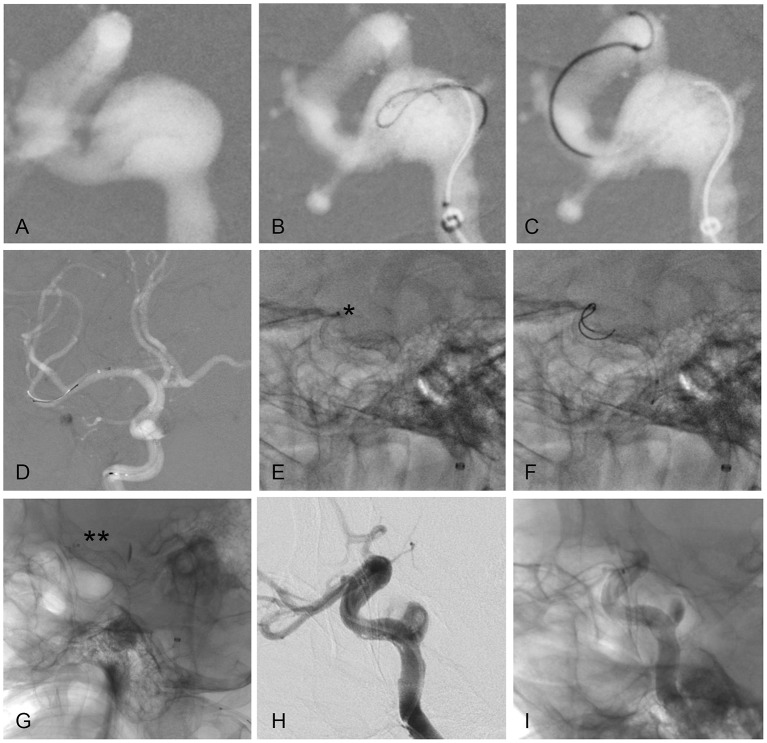
Key steps of the PED rescue maneuver. **(A)** Prolapse of the proximal portion of the stent into the aneurysm sac under roadmap guidance. **(B–D)** A microwire loop is formed in the tip **(B)**, which is then passed through the WCS and distal ICA **(C)** and into the middle cerebral artery **(D)**. **(E)** Insufficient opening of the distal PED (asterisk). **(F,G)** Repeated massage with the microwire facilitated distal PED opening (double asterisks). **(H,I)** Angiography immediately after the procedure revealed that the contrast agent was trapped in the aneurysm cavity, and the typical “meniscus sign” could be seen.

Before the intervention, the patient was started on daily doses of 75 mg clopidogrel and 100 mg aspirin for 5 days. Femoral artery access was set up with a 6-French long sheath (Cook Medical, Bloomington, IN, USA). A 6-French Navien guiding catheter was advanced over a 5-French MPA1 catheter (Cordis, Miami, FL, USA) into the posterior ascending segment of the right cavernous ICA. The roadmap showed that the proximal part of the device had prolapsed into the aneurysm sac (Figure 2A). Angiography revealed that the aneurysm had also become larger. Compared to the previous size, which was 12 × 10 mm with a 12-mm neck, the aneurysm measured 15 × 11 mm with a 15-mm neck.

First, we tried to push the entire WCS into the large aneurysm sac as an alternative rescue strategy. If successful, we planned to deploy a PED along the anatomical aneurysm neck, “imprisoning” the displaced WCS. However, the plan failed, as the WCS was firmly anchored in the distal horizontal segment of the C4 ICA. There were two possible treatments to choose between. Option 1 was to sacrifice the parent vessel. Option 2 was anterograde retrieval of the lost proximal access through the previous WCS.

A 300-cm Transend microwire (Stryker, Kalamazoo, MI, USA) with a loop in the tip was passed through the WCS and distal ICA and into the middle cerebral artery (Figures 2B–D). Subsequently, an XT27 catheter (Stryker) led by the microwire was advanced. An “inside out” approach ensured that the microwire and microcatheter were not caught on the WCS and did not traverse the previous stent. The microcatheter was then withdrawn from the clinoid ICA to the petrous ICA, and a 5.0 × 30.0-mm PED was deployed.

The microwire was advanced again, due to insufficient opening of the distal PED (Figure 2E). Repeated massage with the microwire encouraged distal PED opening (Figures 2F,G). The final angiographic image demonstrated early aneurysm stasis and good PED wall apposition (Figures 2H,I). The patient was awakened from anesthesia and admitted to the intensive care unit.

### Postoperative Course

The patient had an uneventful post-operative course and was discharged in good condition after 3 days. Her oculomotor paralysis improved within 6 months.

## Discussion

The WCS is safe and effective for the treatment of large and giant ICA aneurysms with wide necks (1, 3, 5). Although isolation of the covered stent achieves a high rate of immediate aneurysm exclusion, endoleaks remain a frequent issue after initial covered stent placement. In a prospective multicentre study, Zhu et al. (2) reported that the total immediate aneurysm exclusion rate was 69.2%, with 30.8% undergoing immediate residual endoleaks. Tan et al. (3) reported that 11/19 patients had significant endoleaks in the aneurysm sac, but in nine, they disappeared completely or were significantly reduced after the balloon was reinflated and/or additional covered stent grafts were deployed. As described by Ma et al. (5), 9/57 patients had immediate residual endoleaks, and in two, the leak persisted after performing the remedy. The potential causes of endoleak include non-homogeneous lumens of parent artery diameters between the outlet and inlet vessels, incomplete occlusion of the aneurysm orifice owing to insufficient stent length, and aneurysms located at an acutely angled vessel segment (1, 4). Other causes of endoleak include a wide aneurysm neck, a tear in the graft, and graft recoil after stent deployment (3, 5). In our case, the main contributory factors were insufficient occlusion of the aneurysmal neck by the WCS, a size mismatch between the vascular wall and proximal portion of the stent, and placement at a sharply angled segment. These issues can be resolved by choosing a longer WCS, placing an additional covered stent, and/or re-inflating the balloon. However, in our study, re-inflation of the balloon induced device migration and prolapse into the aneurysmal sac.

Complications related to the migration of the WCS and subsequent prolapse into the aneurysmal sac are rare. However, migration and/or prolapse have been described for intracranial self-expanding stents (6) and also in case analyses or series with PEDs (7, 9). The important risk factors for this phenomenon are short stent length (i.e., the landing zone relative to the aneurysmal orifice was insufficient), large aneurysm size, and the use of multiple devices or percutaneous transluminal angioplasty (PTA) (7, 9, 10). These issues can also contribute to endoleaks. It is very important to measure the diameters of the outflow and inflow vessels accurately. A significant mismatch in the luminal diameter of the device has been related to the “watermelon seed” effect, which may lead to poor stent-to-vessel wall apposition and result in stent migration (7, 9). Hauck et al. (13) described intraoperative PED migration in 2010. The stent shortened, and the proximal portion of the device prolapsed into the aneurysm sac. Access was regained by using the microwire retrograde trans-posterior communicating artery approach. Chalouhi et al. (14) reported delayed migration of a flow diverter device, where the distal end was dislodged into the aneurysm sac. The operator accessed the distal ICA and deployed another PED to salvage it. Martínez-Galdámez et al. (7) described a novel balloon technique for rescue and rearrangement of the proximal part of a PED that migrated into a large aneurysm.

In our case, we believe that there were several main causes of WCS prolapse into the aneurysmal sac: balloon dilatation, which induced stent foreshortening, a short proximal landing zone (<5 mm), robust flow into the aneurysm, and aneurysm location in a tortuous vessel segment. Furthermore, the stent graft formed a flap-like structure with the aneurysm neck. Blood flow into the aneurysm was greater during systole, so the aneurysm gradually increased and would eventually rupture. This increased aneurysm volume and necessitated rapid revision surgery.

Different techniques have been described to solve flow diverter device migration, including open surgery (9), snare-assisted rescue (15), balloon anchoring for distal-end herniation (7, 16), and stent-in-stent techniques (13, 14). Once the flow diverter device has migrated, particularly out of the parent artery lumen with herniation into the aneurysm sac, it is very difficult to use a wire to dislodge the device. The crucial determinant in this situation was whether the migration occurred on the distal or proximal portion of the WCS, since the latter is more challenging. When the distal part of the device prolapses into the aneurysm, there are more endovascular salvage options to regain access. However, when the proximal end is foreshortened, as in our case, the only viable options were trans-circulation rescue or technically challenging anterograde re-access. The most reasonable approach in this case was to deploy another longer PED, overlap the WCS, and redirect the flow jet away from the aneurysm sac. A critical step was passing the C-shaped microwire as a loop across the previously deployed WCS to ensure that the microwire/microcatheter passed through the central axis of the stent, allowing for complete PED opening. Besides, the polytetrafluoroethylene membrane of the WCS may prevent the microwire from inverting, inhibiting the PED from fully opening, which may lead to thromboembolic complications. In our case, a longer PED with a larger diameter was selected to increase stent apposition to the vessel wall and improve system stability. However, as an “off-label” use of the PED, long-term follow-up is needed to evaluate patient outcome adequately.

## Conclusion

Endoluminal vessel reconstruction with a WCS in the treatment of ICA aneurysms is safe and feasible and achieves a high rate of immediate aneurysm obliteration. Endoleaks are a frequent complication that can be resolved by balloon re-inflation and/or placement of an additional stent. However, intra-procedural complications associated with stent migration and subsequent prolapse into the aneurysmal sac occasionally occur. We report the first case of using a PED to successfully realign proximal access to the previous WCS for reconstruction of the parent artery. Preserving proximal access and ensuring that the microwire/microcatheter is securely held within the central axis of the prolapsed stent are critical to achieving complete reconstruction. Besides, accurate stent sizing and optimal vessel wall apposition may minimize the incidence of this unpleasant phenomenon. This is a safe approach that should be considered when a proximal end prolapse of a WCS occurs.

## Data Availability Statement

All datasets generated for this study are included in the manuscript.

## Ethics Statement

Written informed consent was obtained from the individual(s) for the publication of any potentially identifiable images or data included in this article.

## Author Contributions

Z-BW assisted with the procedures, collected data, and drafted the manuscript. SW contributed to manuscript revision. L-GS and X-JY assisted with the procedures. S-QM was the chief performer of procedures and conceived the manuscript.

### Conflict of Interest

The authors declare that the research was conducted in the absence of any commercial or financial relationships that could be construed as a potential conflict of interest.
